# Emerging Therapies for Glioblastoma

**DOI:** 10.3390/cancers16081485

**Published:** 2024-04-12

**Authors:** Stella Aimé Rios, Stephanie Oyervides, David Uribe, Angelica Maree Reyes, Victor Fanniel, Jonathan Vazquez, Megan Keniry

**Affiliations:** School of Integrative Biological and Chemical Sciences, College of Sciences, The University of Texas Rio Grande Valley, Edinburg, TX 78539, USA; stella.rios01@utrgv.edu (S.A.R.); david.uribe01@utrgv.edu (D.U.); angelica.reyes04@utrgv.edu (A.M.R.);

**Keywords:** glioblastoma, EGFR, PI3K, FOXO1, NFLβ, CK2, JAK, STAT3, LIF, oncolytic viruses

## Abstract

**Simple Summary:**

Glioblastoma is the most common malignant brain cancer and is associated with poor prognosis, with an average survival after diagnosis of less than 18 months. Hundreds of clinical trials have been conducted, with more underway to make progress in treating glioblastoma. This review details conventional and emergent targeted therapies for glioblastoma.

**Abstract:**

Glioblastoma is most commonly a primary brain tumor and the utmost malignant one, with a survival rate of approximately 12–18 months. Glioblastoma is highly heterogeneous, demonstrating that different types of cells from the same tumor can manifest distinct gene expression patterns and biological behaviors. Conventional therapies such as temozolomide, radiation, and surgery have limitations. As of now, there is no cure for glioblastoma. Alternative treatment methods to eradicate glioblastoma are discussed in this review, including targeted therapies to PI3K, NFKβ, JAK-STAT, CK2, WNT, NOTCH, Hedgehog, and TGFβ pathways. The highly novel application of oncolytic viruses and nanomaterials in combating glioblastoma are also discussed. Despite scores of clinical trials for glioblastoma, the prognosis remains poor. Progress in breaching the blood–brain barrier with nanomaterials and novel avenues for targeted and combination treatments hold promise for the future development of efficacious glioblastoma therapies.

## 1. Introduction

Glioblastoma is one of the most aggressive cancers. The incidence rate of glioblastoma has risen over the years, with a range of 0.59 to 5 per 100,000 individuals; this is followed by a survival estimate of 12–18 months, with a rate of 5% of diagnosed individuals surviving more than five years [1,2]. Generally, glioblastoma is diagnosed at an advanced stage as it is considered a Grade 4 brain tumor, which limits the efficacy of surgical interventions. Clinical treatments that engage key signaling pathways, together with improved diagnostics, are a crucial requirement for significantly increasing the survival rate for glioblastoma patients.

This review includes a broad overview of glioblastoma therapies, including anti-neoplastic drugs such as temozolomide (TMZ), nanomaterial delivery systems, oncolytic viral therapy, and targeted therapies [3,4,5,6,7,8,9,10]. The novelty of this review is that it provides a broad overview of the leading translational strategies that act on major signaling pathways, especially for researchers with less background in glioblastoma therapeutics. Other reviews offer more in-depth analyses for individual classes of glioblastoma treatments, such as targeting PI3K (phosphatidylinositol 3 kinase) [11]. This review includes a discussion of key signaling pathways in glioblastoma, delineating how these pathways were targeted in this aggressive cancer, as well as patient outcomes. Pathways discussed include EGFR (Epidermal growth factor receptor), PI3K, NFκβ (Nuclear factor kappa-light-chain-enhancer of activated B cells), JAK-STAT (Janus Kinase- Signal transducer and activator of transcription), CK2 (Casein kinase 2), WNT (wingless-type MMTV integration site family), NOTCH, Hedgehog and TGFβ (transforming growth factor beta) [12,13,14,15,16,17,18]. Although most signaling pathways for glioblastoma are identified, targeting these pathways in clinical trials has not substantially improved outcomes. These pathways are highly interconnected and have similar impacts on glioblastoma, such as promoting stem characteristics, invasiveness, and therapeutic resistance. One significant barrier to effectively treating glioblastoma is overcoming redundancy issues regarding targeted therapies to these pathways. The degree to which EGFR, PI3K, NFKβ, JAK-STAT, CK2, WNT, NOTCH, Hedgehog, and TGFβ are dependent on each other, and redundant with each other, in the context of glioblastoma therapeutics remains to be fully elucidated. The next horizon for targeted therapies will be to determine which drug combinations are synthetically lethal for glioblastoma. Another major barrier to treating glioblastoma is drug delivery to the tumor. We include a discussion of nanomaterials as carriers for chemotherapies to glioblastoma tumors. One last barrier is leveraging immunotherapies with targeted therapies for glioblastoma to promote immune cell infiltration to the tumor and effectively initiate apoptosis. Combined immunotherapy and pathway-targeted therapy trials are underway and hold the greatest promise for enhancing glioblastoma treatment.

## 2. Anti-Neoplastic Drugs and Surgical Interventions for Glioblastoma

### 2.1. Temozolomide (TMZ)

In the 1940s, DNA alkylating agents were discovered to have anti-tumorigenic effects, leading to the development of the first class of chemotherapeutic drugs [5]. One of these alkylating agents, temozolomide (TMZ), was synthesized for medical use in the late 1970s, gaining clinical approval for usage in the United States and Europe in the early 2000s [5]. As an imidazotetrazine lipophilic prodrug, TMZ can cross the blood–brain barrier (BBB), becoming physiologically activated by the body’s pH, which then allows TMZ to convert into the metabolite 5-(3-methytrazen-1-yl)imidazole-4-carboxamide (MTIC) [5].

Once MTIC has become activated within the body, it is hydrolyzed to produce methyldiazonium ions. These methyldiazonium ions then methylate DNA at the O6 position of guanine, forming several DNA adducts. The formation of these DNA adducts then triggers DNA damage, leading to cytotoxicity within the cell [5]. As DNA replication and transcription in cancer cells halt, apoptosis is induced. TMZ is a first-choice chemotherapeutic agent for glioblastoma.

Over 50% of glioblastoma patients do not respond to therapy. One of the main contributors to this obstacle, O6-methyguanine-DNA-methytransferase (MGMT), is crucial in counteracting DNA alkylation damage and maintaining repair activity when triggered [5]. MGMT’s restorative activity can nullify the cytotoxic effects of TMZ by blocking methylation at the O6-guanine position of DNA [5]. The epigenetic landscape of glioblastoma is impacted not only by MGMT, but also *IDH1* and *IDH2* status [19]. *IDH1* mutation alters the epigenetic landscape and is associated with a more favorable outcome in glioblastoma [19,20,21]. Mismatch repair and base excision repair mechanisms also impact the efficacy of TMZ therapy as repairing alkylating adducts would hinder drug treatments [22]. Tumor suppressor p53 status also plays a key role in the efficacy of TMZ treatment for glioblastoma [22].

Furthermore, dysregulation of common cancer signaling pathways also contributes to TMZ resistance. Research has shown that dysregulation of the PI3K/AKT pathway occurs in a significant percentage of glioblastoma tumors [5]. Known for promoting chemoresistance, the PI3K/AKT pathway inhibits apoptosis and promotes cellular survival through proliferation and angiogenesis via activating anti-apoptotic proteins, negating the effects of TMZ [5,15].

In addition, dysregulation of both the canonical and noncanonical pathways of WNT contributes to chemoresistance, promoting tumor survival of glioma via stem cell maintenance, cell proliferation, and invasion. The loss of DOC-2/DAB2 interacting protein (DAB2IP) leads to TMZ resistance [6]. This loss of function leads to the suppression of TMZ-induced autophagy via the negative expression of autophagy-related protein 9B, ATG9B, a vital protein involved in tumorigenesis and cellular homeostasis [6].

### 2.2. Carmustine

Another alkylating agent, carmustine (a nitrosourea derivative, {BCNU, [1,3-bis (2-chloroethyl)-1-nitrosourea]) can be employed as a single agent anti-neoplastic therapy or as a bridge therapy between surgery and TMZ [23,24,25]. Carmustine is delivered intravenously or directly to the site of tumor resection in a biodegradable form such as the Gliadel wafer [23,24]. Treatment with carmustine extends survival by two to four months [23,26]. However, side effects with carmustine treatment were noted, such as nausea, vomiting, hematotoxicity, and pulmonary fibrosis [27]. Carmustine is reported as less effective than TMZ as a single agent [28]. Trials using carmustine in combination with anti-angiogenic drug bevacizumab had promising results [29,30].

### 2.3. Surgical Resection

Over the years, researchers concluded that the safe removal of malignant gliomas is associated with higher patient survival outcomes. As the standard of care, surgical resection remains a common option when considering a glioblastoma tumor. Diagnostic imaging, such as magnetic resonance imaging (MRI), is utilized to identify the location and determine the severity of the glioma [2,31]. One significant advantage of MRI is that ionizing radiation is not used, unlike a computed tomography (CT) scan [2].

Furthermore, maximally safe surgical resection is the primary goal of this therapy, aiming to remove as much of the glioma as possible to alleviate symptoms for the patient. However, based on the anatomical structure and severity of the glioma, surgical resection may not be a feasible option when considering neurological function [2,5]. Due to its invasive nature, high-grade gliomas are more commonly found surrounding the brain parenchyma, posing an obstacle for neurosurgeons as removing the glioma would simultaneously damage surrounding brain structures [2,32].

### 2.4. Fluorescent-Guided Surgical Resection

As of today, the most sensitive approach for precisely resecting glioblastoma cells is through fluorescent-guided surgical resection, which involves the use of 5-aminolevulinic acid (5-ALA), which specifically targets the glioma, illuminating the cancerous tissue at a particular wavelength and distinguishing it from normal brain tissue [31,33,34]. Fluorescent-guided resection can differentiate cancerous glioma tissue that would otherwise appear normal on an MRI scan [31,32,34]. When comparing fluorescent-guided resection with standard white light resection, glioblastoma patients treated with fluorescent-guided resection showed a significant decrease in tumor proliferation at six months [2,31].

Additionally, sodium fluorescein can be used during fluorescent-guided resection of glioblastoma. Although not specific to malignant glioma cells, it has a significant advantage of identifying BBB impairment by accumulating at breaches [31,35]. As it accumulates at the borders of a glioma, it becomes easily visible on an MRI scan. This allows surgeons to differentiate between healthy tissue and tumor tissue. Compared to 5-ALA, sodium fluorescein is often preferred due to its cost-effectiveness and manageability, available to administer immediately and without significant side effects [31,35].

## 3. Tumor Treating Fields (TTFields)

As one of the more innovative forms of therapy, tumor-treating fields (TTFields) involve low-intensity electrical fields that interrupt cell division, inhibiting tumor growth with its anti-proliferative effects [36]. The FDA approved TTFields in 2015, which has shown great potential as a non-invasive treatment option for glioblastoma patients [36]. Patients who received TTField treatment for glioblastoma displayed an overall survival median of 22.6 months as opposed to 17.4 months for patients who did not undergo TTField treatment [36]. Additionally, recent clinical trials combined TTFields with TMZ, demonstrating a significant increase in the overall survival of glioblastoma patients, generating a median overall survival of 20.9 months compared to the approximate 15-month survival alone [36]. In addition to this advantage of TTField therapy, patients responded well to treatment with minimal side effects and high tolerance [36].

## 4. Blocking Angiogenesis in Glioblastoma

Glioblastoma tumors are heavily vascularized, making anti-angiogenic agents ideal to target this cancer [37,38,39,40,41,42,43]. Bevacizumab (Avastin) is a humanized monoclonal antibody that binds to VEGF to neutralize its action to prevent blood vessel growth. Bevacizumab treatment extends progression-free survival of glioblastoma but does not extend overall survival [44]. There are over two hundred clinical trials examining bevacizumab therapy in glioblastoma, including current trials using super selective intraarterial cerebral infusion of this drug to target tumors, studies with anti-neoplastic drugs such as TMZ, and studies with targeted therapies such as TORC1/2 Inhibitor MLN0128 [45,46,47]. One promising drug combination includes bevacizumab with alkylating agent lomustine, which was found to increase glioblastoma overall survival [48]. Treatment with bevacizumab includes side effects such as fatigue, hypertension, and proteinuria. Bevacizumab toxicity is a significant consideration in employing this therapy to treat glioblastoma [37,38,39,40,41,42,43,44,45,46,47].

## 5. Targeting EGFR in Glioblastoma

Approximately 50% of glioblastoma tumors have amplified *EGFR*, which is associated with a worse prognosis [49]. *EGFR VIII* mutation encodes an activated receptor tyrosine kinase and is found in 25–64% of glioblastoma [50,51]. Given the importance of EGFR in the development and progression of glioblastoma, it is an attractive chemotherapeutic target [52,53,54,55,56,57,58,59]. However, small molecule inhibitors and antibody-based therapies in the clinic demonstrate little to no therapeutic benefit in treating glioblastoma, Table 1. This contrasts with non-small cell lung cancer, where *EGFR* is commonly mutated in the tyrosine kinase domain and can be effectively druggable with ATP-site competitive inhibitors [60,61]. The extracellular domain-mutated *EGFR VIII* found in glioblastoma is not effectively targeted by the ATP-site competitive inhibitors, potentially owing to other receptor tyrosine kinases (RTKs) such as MET (mesenchymal-epithelial transition factor) and PI3K bypassing the requirement of EGFR catalytic activity [62]. The BBB impacts small molecule and antibody therapeutic efficacies for glioblastoma. Nanomaterials and drug optimizations that overcome the BBB hold promise in targeting EGFR for glioblastoma treatment [63].

Effectively targeting EGFR in glioblastoma will require a dramatic shift in approach. Once drugs are effectively delivered to the tumor site, which requires breaching the BBB, the agent must block EGFR output. Simply inhibiting the tyrosine kinase activity is insufficient to block EGFR action in glioblastoma, as TKIs (tyrosine kinase inhibitors) fail to reduce downstream signaling in clinical trials [56,57,58]. Moving forward, science must discern the contributions of EGFR independent of its tyrosine kinase activity and redundant pathways that restore Ras and PI3K in the context of EGFR inhibition.

## 6. PI3K Is a Therapeutic Target for Glioblastoma

The PI3K Pathway is almost always activated in glioblastoma [64]. In adult and pediatric glioblastoma, the gene encoding the catalytic subunit of PI3K (*PIK3CA*) is mutated to an active form approximately 20% of the time [64]. The tumor suppressor gene *PTEN* encodes a phosphatase that performs the reverse reaction of PI3K and is mutated in 30–40% of glioblastoma [65,66]. Clinical trials targeting PI3K with small molecule inhibitors are underway. BKM120 (Buparlisib) is a pan PI3K inhibitor that overcomes the BBB and is well tolerated [67,68,69]. As a single agent, BKM120 has minimal impact on glioblastoma [68]. The combination of low-dose BMK120 and bevacizumab was not well tolerated in glioblastoma clinical trials [70]. The ATP-competitive dual PI3K and mTORC1/2 inhibitor, NVP-BEZ235, reversibly inhibits AKT activation and is utilized in clinical trials, including for glioblastoma [67,71]. To date minimal to no benefit on glioblastoma has been observed with NVP-BEZ235 treatment [67]. Although the PI3K Pathway drives glioblastoma, targeting this pathway is challenging due to toxicities and feedback mechanisms that bypass drug interventions [72].

## 7. Targeting FOXO1 in Glioblastoma

FOXO1 drives stem gene expression in embryonic stem cells and glioblastoma [73,74]. Inhibition of FOXO1 with AS1842856 robustly induced apoptosis in LN229, A172, LN18, U118MG, and DBTRG glioblastoma cell lines, highlighting a novel targeted therapy that disrupts circuitry required for cancer progression [75]. Perhaps targeting FOXO1, a transcriptional driver of stemness in glioblastoma, will prove more effective in the clinic than other therapies as the cancer stem cells are strongly associated with poor prognosis [76,77,78]. Glioblastoma cancer stem cells (GSCs) are associated with chemoresistance, increased invasiveness, and increased tumor plasticity [78]. Therapies directed at cancer stem cells may be vital in blocking glioblastoma progression.

## 8. The NF-κB Pathway as a Glioblastoma Therapeutic Target

NF-κB is largely known for the immune response associated with the signaling pathway. However, NF-κB commonly displays aberrant signaling in glioblastoma [79]. This aberrant signaling has been associated with several tumor-related activities, including growth, invasiveness, and increased resistance to chemotherapies. NF-κB is activated through several stress signals in cancer, including ROS, growth factors, and DNA damage [80]. These signals lead to elevated signaling levels in the pathway and often contribute to favorable conditions for oncogenic growth.

The NF-κB Pathway initiates through several extracellular receptors, including the EGFR and the Transforming Growth Factor Beta Receptor (TGFβR) superfamily that bind to various cytokine factors secreted during immune responses [13,81]. The NF-κB1/2 proteins are transcription factors that are the terminal products of both the canonical and noncanonical pathways and, in cancer, are known to target several genes associated with increased oncogenesis and decreased apoptosis. In the tumor microenvironment (TME), cytokines are secreted at elevated levels, leading to an elevated level of NF-κB1/2 protein and aberrant activation of the pathway. In cancerous conditions, we see highly elevated levels of NF-κB1 and NF-κB2 proteins present in the nucleus and mitochondria of the cell, while under normal conditions, these proteins are localized primarily to the cytosol and highly targeted by ubiquitin–proteasome degradation [82]. Future directions for treating NF-κB in glioblastoma may lie in studying its interaction with proteins in the mitochondria [82].

Current research into glioblastoma treatment through NF-κB inhibition has shown promising results. Through treatment with TMZ (the current gold standard of glioblastoma chemotherapy) and NF-κB BAY 11-7082, researchers have shown increased apoptosis in patient-derived glioblastoma cell lines [83]. Combining these drugs resulted in the slowed migration of lab-generated tumors versus the activity of the two drugs independently. The preliminary data also suggested that these drugs with co-treatment may impact PI3K/AKT/mTOR/NF-κB crosstalk. Some preliminary data indicates that inhibiting key kinases from the NF-κB pathway impacts glycolysis/OXPHOS within glioblastoma cells and negatively impacts the growth of tumors in mouse xenograft models under a ketogenic diet [84]. Another potential avenue that these findings open is the study of NF-κB and mitochondrial metabolism as it relates to this cancer. Glioblastoma being more reliant on OXPHOS metabolism than most cancers makes it more susceptible to OXPHOS inhibition, and these findings suggest that more could be understood about the exact mechanism by which NF-κB impacts this metabolic switch within the mitochondria [85].

## 9. Targeting CK2 in Glioblastoma

### 9.1. CK2 Biology

In the 1950s, Casein Kinase 2 (CK2) was identified due to its ability to phosphorylate casein, a protein most commonly found in milk [86]. Over 500 known substrates of this kinase are estimated to be responsible for 10% of the human phosphoproteome [86]. Since then, further research has led to its crucial role as a serine/threonine kinase in multiple cellular processes vital to the survival of a cell, such as involvement in signaling pathways, regulating transcription factors, and managing regulatory proteins [86,87,88]. Overexpression of CK2 in various cancers, such as glioblastoma, leukemia, lung cancer, breast cancer, and melanoma, increases cell proliferation and metastasis [86,89,90].

### 9.2. Biological Functions of CK2 in Glioblastoma Cancer

CK2 is highly expressed in glioblastoma, creating a favorable cellular environment for cancer development through modulating cell proliferation, cell invasion, metastasis, angiogenesis, and resistance to apoptosis [86,88,89]. In the tumor microenvironment of glioblastoma, CK2 influences the cytoskeleton, microtubules, ion channels, and extracellular matrix components [86,91]. CK2 can also rewire energy metabolism, leading to altered cell proliferation.

### 9.3. Inhibition of CK2 *via* CX-4945 as a Glioblastoma Therapy

Introduced as the first clinical-stage CK2 inhibitor in 2010, CX-4945 has been researched as a targeted approach to treat several malignancies and disorders, including leukemias, lymphomas, breast cancer, glioblastoma, and even cystic fibrosis [92,93,94]. A phosphoproteomic study using mitotic HeLa cells confirmed that CX-4945’s primary kinase target is CK2 [95]. CX-4945 inhibits CK2 by binding to its ATP-binding site, disrupting phosphorylation and preventing the activation of signaling proteins. Moreover, CX-4945 induces apoptosis and inhibits cancer stem cells, showing promising antitumor effects [95].

Preliminary research has demonstrated CX-4945’s substantial antitumor effects, such as promoting cell cycle arrest and apoptosis in cancer cell lines; moreover, preclinical research showcases the antitumor benefits of CX-4945’s suppression of CK2, shown to be effective in treating glioblastoma, leukemia, and breast cancer, among other cancers, Table 2 [94,96,97,98]. It has been determined that these anti-cancer effects are a direct result of the inhibition of CK2, heavily supported by the substantial decrease in phosphorylation of primary substrates seen within a few hours after CX-4945 treatment [9,95,99].

Studies have shown that inhibition of CK2 can also impede cell migration in glioblastoma [95,99,100]. GL261 cells were then treated with TMZ, CX-4945, APG, TBB, and TDB, and the cell viability was determined after 72 h of incubation with the respective drug [101]. This led to additional in vitro experiments evaluating the combined treatment of CX-4945 and TMZ to cultured GL261 cells. Administered alone, TMZ reduced cell viability to 82.8% ± 5.6% at 1 mM and 59.2% ± 3.2% at 1.5 mM, whereas CX-4945 reduced cell viability to 52.0% ± 1.4% at 30 μM and 31.9% ± 2.1% at 50 μM [101]. When both therapeutic agents were combined, the results surpassed the efficiency of each drug alone, showcasing a 35.6% ± 4.7% (TMZ 1 mM + CX-4945 30 μM) and 21.5% ± 1.0% (TMZ 1.5 mM + CX-4945 50 μM) reduction in cell viability [101].

When used with other anti-cancer medications, CX-4945 has demonstrated synergistic results. In some treatments, other kinase inhibitors, such as EGFR and PI3K/AKT/mTOR inhibitors, are combined with CK2 inhibitors [95,101,102]. These inhibitors could target glioblastoma cancer cells by inducing autophagy and improving the efficacy of current medicines, either by themselves or in combination.

**Table 2:** Treatment of glioblastoma cells with CK2 inhibitors.

Drug Name	Context	Impact
CX-4945 treatment alone and with TMZ	Mouse xenograft (GL261) and in vitro methods	Apoptosis induction, inhibition of cell migration, adhesion, and colony formation, inhibition of STAT3, NF-kB p56, and Akt; CX-4945 in combination TMZ had more robust impact [101]
CX-4945 treatment w/combined inhibitors: gefitinib and TMZ	In vivo mouse xenografts (GL261, SF767, U373, LN229) and in vitro methods	Inhibition of cell proliferation; CX-4945 sensitized TMZ-resistant cell lines (SF767) [101,103]
CX-4945 treatment w/combined inhibitor gefitinib	In vivo mouse xenograft (Xenograft X1046) and in vitro methods (X1016, X1046, X1066, U251-MG, U87MG)	Inhibition of tumor growth, cell proliferation, migration, adhesion; decreased activation of STAT3, NF-kB p56, and Akt; reduced stemness [104,105]

### 9.4. Limitations of CX-4945 Therapies

As with any cancer therapy, there will be side effects. One study highlighted CX-4945’s high micromolar concentration to induce cell death in cancer cells—this may have certain implications when looking at CX-4945 from a clinical point of view [95]. Along with these disadvantages, there is a possibility that cancer patients may develop a resistance to CX-4945 treatment, which will lead to the obstacle of finding another therapy that will remain effective in inhibiting cancerous growth.

## 10. Targeting JAK/STAT in Glioblastoma

The JAK/STAT Pathway plays a major role in many biological functionalities, such as cell growth, cancer development, proliferation, and metastasis. Cytokine receptors are bound by ligand, leading to Janus tyrosine kinase activation. JAK1, in turn, phosphorylates and activates STAT3, which drives the expression of pluripotency targets such as *OCT4* and *SOX2*, as well as leukemia inhibitory factor (LIF) [106].

In glioblastoma, the JAK/STAT Pathway promotes tumorigenesis (Table 3). Ruxolitinib, a JAK inhibitor, reduced glioblastoma invasiveness and tumorigenesis. With dosages ranging from 50–200 nM in 24 treatments, 200 nM Ruxolitinib had significant tumor inhibition. Another study found that 195 nM Ruxolitinib inhibited glioma invasion via disruption of the IL-6 dependent JAK2/STAT3 pathway [107].

SAR317461, a known JAK2 inhibitor, inhibited STAT3 phosphorylation but not STAT5. Treatment concentrations of 10 μM and 2 μM rendered STAT3 phosphorylation undetectable. This allowed for induced cell autophagy and enhancement of apoptosis. With this mechanism, pSTAT3 may need to be present for potent SAR317461-associated anti-glioblastoma activity [108].

WHI-P131 and PF-956980 JAK3 inhibitors, were researched and found to effectively reduce glioblastoma proliferation rates but not induce cell death. With JAK3 inhibitors, DNMTs (DNA methyl transferases) were highly expressed compared to untreated cell lines. These findings helped suggest that JAK3 inhibitors could alter glioblastoma epigenetics, preventing possible maturity and proliferation [109].

**Table 3:** Targeting JAK-STAT in glioblastoma.

Drug Name	Context	Impact
Ruxolitinib	Human glioblastoma cells, U87MG	Ruxolitinib inhibited IFNs (IFN-α and IFN-γ)-dependent JAK/STAT signaling, decreased invasiveness and tumorigenesis [107]
Ruxolitinib	Human glioblastoma cells, U87MG	Inhibits IL-6 receptor-dependent JAK/STAT signaling (IL-6/JAK2/STAT3 axis) significantly inhibited tumor invasion (95.2%) [110]
JAK2 inhibitor SAR317461	Human U87MG, U251 and A172 Glioblastoma	Potently inhibited STAT3 phosphorylation (10, 2, and 0.1 µM [108]

## 11. Targeting LIF in Glioblastoma

Leukemia inhibitory factor (LIF) is a pleiotropic cytokine and a member of the IL6-type cytokine family [111,112,113,114]. It plays a significant role in many biological functions, such as prompting differentiation, cell growth, cancer development, proliferation, metastasis, etc. Furthermore, LIF has been proven to support self-renewal cancer cells, induce tumorigenicity, and assist in resistance [115]. LIF regulates signaling pathways such as the JAK/STAT3, PI3K/AKT, and SHP2/MAPK

Small-molecule inhibitors that target LIF/LIFR, such as EC359 and EC330, were used to treat ovarian cancer (OCa), breast cancer, and endometrial cancer (ECa), which inhibited tumor development in OCa cells, ECa cells, and breast cancer MCF7 cells [111,112,113,114,116]. Nasopharyngeal carcinoma cells in vitro and xenograft mouse studies were treated with soluble LIF receptors, LIF antagonists (decreases LIF-mediated effects), or mTOR inhibitors, leading to growth arrest and increased sensitivity to gamma radiation [117]. Targeting LIF (both vivo and vitro, including mouse models) reduced cell viability, migration, survivability, tumor growth, and proliferation in breast cancer [116] and triple-negative breast cancer [113], while in type II endometrial cancer [111], tumor growth significantly decreased. MSC-1 [113], LIF receptor (sLIFR) [117], and LIF neutralizing antibodies [118] demonstrate the ability to effectively block LIF from binding LIFR, including other cytokines. They reduced cell viability, migration, survivability, tumor growth, and proliferation in ovarian cancer, breast cancer, endometrial cancer, and triple-negative breast cancer. Despite the positive results, there are drawbacks, such as increased toxicity levels, surviving tumor cells adapting to therapeutic agents, combined therapies negating each other, and therapeutic targets still being studied and considering that these LIF targeting inhibitors or antibodies could be utilized in glioblastoma [52,111,112,113,114,115,116].

## 12. WNT Pathway in Glioblastoma

The WNT Pathway plays crucial roles during embryonic development and in tissue homeostasis [119]. The WNT Pathway sustains stem cells, promotes invasion, and contributes to TMZ resistance in glioblastoma, making it an ideal drug target for this cancer. Recent studies highlight another extremely key role for the WNT Pathway with reference to the BBB [6,119,120]. During development, the WNT Pathway establishes proper vascular architecture, including the BBB. Murine models with disruption of this pathway in the central nervous system (genes *Ctnnb1*, *Lrp5*, and *Lrp6*) harbor leaky blood vessels with hindered BBB development [120,121]. Clinical trials with non-steroidal anti-inflammatories such as celecoxib were evaluated for enhancement of chemotherapy in glioblastoma with no added benefit [122,123,124]. Small molecule inhibitors to the WNT pathway have been investigated in cell culture studies, and murine models with noted decreases in tumor size, cell proliferation, and infiltration [125,126,127].

## 13. Targeting the NOTCH Pathway in Glioblastoma

The NOTCH Receptors (1–4) are evolutionarily conserved and play critical roles during development [128,129]. The roles of NOTCH1 and NOTCH2 promote glioblastoma growth and invasion in cancer cell lines such as U251, 5310, A172, U87MG, LN18, and LN229 [128,129,130,131]. NOTCH binds to the extracellular ligand, leading to cleavage events that ultimately release an intracellular domain that serves as a transcription factor [128,129]. Numerous clinical trials that target the NOTCH Pathway, particularly NOTCH cleavage in glioblastoma, were carried out. Gamma secretase inhibitor RO4929097 has no therapeutic benefit in recurrent glioblastoma patients and failed to inhibit neurosphere formation in fresh tissue samples from glioblastoma patients [132]. The combination of RO4929097 with TMZ and radiotherapy was well tolerated. However, RO4929097 had variable BBB penetration [133]. Therefore, although the NOTCH Pathway promotes cancer stem properties and glioblastoma cell proliferation, targeting this pathway is challenging due to the BBB. Even in tissues from glioblastoma patients, targeting NOTCH with a gamma-secretase inhibitor had limited efficacy, suggesting alternate pathways can bypass the NOTCH requirement, at least in some tumors [133].

## 14. Hedgehog Signaling Pathway as Putative Glioblastoma Target

The Hedgehog Pathway is active during embryonic development to pattern the central nervous system, lungs, teeth, hair follicles, and other tissues [18,134]. The Hedgehog pathway is also activated during wound healing. Ligands such as sonic hedgehog (SHH) binds to the patched receptor, ultimately leading to transcriptional activation via GLI family transcription factors such as Glioma-associated oncogene 1 (GLI1) [135]. Clinical trials to disrupt this pathway are still underway. For example, the SHH receptor can be targeted with the drug ABTC-0904 [136]. However, to date, there has been limited therapeutic benefit to targeting the Hedgehog Pathway in glioblastoma, potentially due to redundant pathways [137].

## 15. Targeting the TGFβ Pathway in Glioblastoma

The TGFβ Pathway is crucial for embryonic development and proper regulation of inflammatory responses [138]. The role of TGFβ in cancer depends on the context. It can lead to a cytostatic response or promote drug resistance, angiogenesis, and stemness in more advanced cancers [138,139]. Secreted TGFβ ligand binds to heteroduplexed TGFβRI and TGFβII receptors. In response to TGFβ, type I receptors phosphorylate the transcription factors SMAD2 and SMAD3, leading to an interaction with SMAD4 and transcription [140]. Targeting the TGFβ Pathway is extensively studied in clinical trials for cancer, including glioblastoma. Small molecule inhibitors such as Vactosertib and Galunisertib, neutralizing antibodies such as Fresolimab, ligand traps such as Bintrafusp, and anti-sense nucleotides such as Trabedersen were examined in completed clinical trials with little added survival benefit for glioblastoma [140]. There are ongoing clinical trials that examine the efficacy of combined TGFβ inhibition with immunotherapies and/or anti-neoplastic drugs [140].

## 16. Oncolytic Virotherapy’s Potential Role in Glioblastoma Multiform Prognosis

As the most prevalent high-grade brain tumors, glioblastomas have the lowest survival rates as they are unresponsive to conventional oncological treatments [141]. Therefore, other possible therapies have begun to be researched—such as the applicability of immunotherapeutic treatments, more specifically, the use of oncolytic viruses/virotherapy, which has been extensively studied for the past two decades, showing promising results in clinical trials [142]. Within this period, research into fifteen oncolytic virus families has been analyzed, nine of which have been included in clinical trials [143]. Two of the nine viruses, Herpes Simplex Virus Type I (HSV-1) and DNX-2401, are included in this review, including clinical trial data and overall effectiveness analysis. As this treatment option is new, further research must be conducted to combat the limitations present. Therefore, in this review, we discuss the applicability and methodology associated with employing various oncolytic viruses as immunotherapy against glioblastomas and address the limitations of this treatment.

### 16.1. Oncolytic Virotherapy

Throughout the past century, oncolytic virotherapy has been utilized as oncological treatment as preliminary research has shown that introducing viruses into cancer patients has led to remission [141]. Although this treatment is typically employed in leukemia or lymphatic cancers, clinical trials have begun to test its applicability in glioblastoma [141]. Glioblastoma is an adequate candidate for oncolytic virotherapy as these tumors do not metastasize and localize specifically to the central nervous system [141]. Employment of viruses permits the termination of cancer cells only, allowing healthy cells/tissues to remain unaffected [144]. Immunogenic cell death (ICD) ensures the death of these malignant cells through apoptosis, autophagy, or necrosis by encouraging antigen-presenting cells to travel along the lymphatic system to recruit CD8+ T lymphocytes. These cells ultimately cause the death of cancerous cells [145]. Due to the BBB in glioblastomas, viruses must be modified to overcome this barrier, depending on the identity of the virus [145]. These modifications, for example, include using convection-enhanced delivery (CED) of a nonpathogenic polio-rhinovirus chimera [145]. Administration through CED permits the virus to be delivered safely and effectively to the interstitial spaces in the central nervous system while improving the chances of overcoming the BBB, as the delivery is completed intratumorally [145]. Although CED overcomes the BBB, individual considerations on the delivery method must be considered depending on the virus. Despite noted limitations, oncolytic virotherapy maintains a promising potential treatment option for glioblastoma, as engineered oncolytic virotherapy strains encourage precise tumor treatment and immune response [143].

### 16.2. Herpes Simplex Virus Type 1

Special concentration has been placed on genetic engineering HSV-1 over the years to test its efficacy as a potential oncolytic virotherapy option. Although, typically, the utilization of this virus in neural tissue can be fatal, researchers derived a conditional replicating HSV-1 strain known as G207 [146]. Through clinical trials, the G207 strain has been denoted as a potential model due to its high efficacy in vitro and in vivo gliomas [146]. Multiple clinical trials show that viruses can be mutated to conform adequately to glioblastoma treatment, as seen in HSV and many other viruses analyzed. In research conducted by Markert et al., glioblastoma patients underwent a trial phase that provided them with the mutagenic strain of HSV-1, G207, in a dose escalation process [146]. Amongst the twenty-one patients, all were screened for having previously undergone external beam radiotherapy. All patients were categorized according to age, gender, tumor location, and diagnosis date. Additionally, within this trial, it is essential to note that four of the twenty-one patients had anaplastic astrocytoma, not glioblastoma. Before undergoing the surgical procedure, the patients obtained a contrast-enhanced CT scan to ensure maximal localization of the glioblastoma tumors [146]. G207 was injected into each patient’s tumors during the surgery. The tumor volumetrics post-treatment per patient were then analyzed through MRI evaluations and survivability length [146]. Through the prior analytical methods mentioned, the mean amount of time between treatment and death amongst deceased patients was determined to be 6.2 months, while four patients lived over 12.8. The researchers concluded that dosages of up to 3 × 10^9^ p.f.u. were safe to inoculate into brain tumors. Antitumor activity was present when HSV-1 was administered to glioblastoma patients. Despite the adverse effects noted in some patients, the utilization of viruses for glioblastoma treatment is still a viable option [146].

In research conducted by Todo et al., HSV-1, titled G47Δ, is triple mutated and used as a treatment option for those with recurrent glioblastoma [147]. This strain of HSV-1 was delivered twice to patients intratumorally at varying dosages of either 3 × 10^8^ pfu or 1 × 10^9^ pfu. Among the twelve patients in the trial, all but one individual experienced adverse effects [147]. Median survival seen among the patients was 7.3 months, and only 38.5% of the patients reached the one-year mark [147]. Out of the thirteen patients, however, three individuals lived over forty-six months [147]. Within phase I of the trial and through biopsies, MRIs, and the survivability rates seen among the patients, it is evident that G47Δ is an effective strain to use as a treatment against the glioblastoma multiform [147]. Within Phase II of Todo et al., nineteen adult patients suffering from supratentorial glioblastomas were analyzed. Amongst these patients, all underwent radiation therapy, TMZ, and six doses of G47Δ that were administered intratumorally [147]. Within this second part of the trial, the overall survival median was 20.2 months after G47Δ administration and 28.8 months after the primary surgery that was conducted [147]. Due to the increment in survivability seen within the two phases and the minimal adverse effects experienced by patients, G47Δ was approved as the first oncolytic virotherapy in Japan [147].

Contrasting other clinical trials conducted on oncolytic Herpes Virus Strains, the trial conducted by Ling et al. looks at CAN-3110, a different HSV-1 strain that retains ICP34.5, a neurovirulence gene [148]. ICP34.5 is driven by the *NESTIN* promoter, commonly overexpressed in glioblastoma tumors, and is known to play a role in neuropathogenicity [76,148]. This trial obtained a median overall survival rate of 11.6 months among forty-one patients with HGG/glioblastoma [148]. The survivability seen in the patients was related to the individuals having an HSV1-positive serology before the CAN-3110 employment and an increase in T cell frequency and tumor transcriptomic signatures that were both present after the treatment [148]. Overall, the employment of this strain provides evidence of the effectiveness seen in intralesional oncolytic virotherapy when treating glioblastoma [148].

### 16.3. DNX-2401 Clinical Trials

Presented as an option to treat glioblastoma, DNX-2401 is an oncolytic virus that was engineered to undergo conditional replication, specifically targeting cancer cells [149]. Administration of this adenovirus to patients with high-grade gliomas as an antitumor treatment was proven to be safe and effective [149]. The clinical trial consisted of forty-nine patients treated with a singular dose of DNX-2401 and pembrolizumab, a standard cancer immunotherapy [149]. When considering the minimal adverse effects of this treatment, patient survivability was extended to a median of 12.5 months, with some patients exceeding this average with a survivability of over 60 months [149]. The survivability seen at the twelve-month mark was met by 52.7% of the individuals; meanwhile, the control had a rate of 20%. Ultimately, using DNX-2401 as a glioblastoma treatment option was effective when treated alongside pembrolizumab as it depicted an anti-tumoral response with minimal adverse effects among patients when the two treatments were used together [149]. The success experienced in this virus employment led it to receive the FDA’s fast-track designation [150].

### 16.4. Limitations of Oncolytic Viral Therapy for Glioblastoma

A contributing factor to the poor prognosis experienced by glioblastoma patients is due to the presence of the BBB in the central nervous system [151]. As the blood–brain barrier acts as an immune system extension that protects the brain from pathogens, the effectiveness of oncolytic therapeutics is limited [76]. Therefore, when considering treatment options, tumor-specific signaling pathways are a reasonable target for researchers to examine [151]. Through clinical trials, it is evident that by doing so, antitumor responses are being obtained as the BBB is being effectively penetrated by treatments [151]. The decrease in limitation experienced by the blood–brain barrier allows for immunotherapeutic treatments, such as oncolytic virotherapy, CAR T cell therapy, and vaccination therapy, to be a blossoming field in glioblastoma treatment, research, and development [141].

## 17. Nanomaterials to Treat Glioblastoma

The BBB is a significant obstacle for chemotherapeutic delivery to glioblastoma tumors [7,8]. Nanoparticles hold promise in breaching the BBB with a diverse range of structures, including polymeric, micellar emulsions, and inorganic particles such as gold particles [7,8]. Nanoparticles also can have the added benefit of enhancing drug efficacy beyond the impact of the drug alone [152]. Nanoparticles can aid in delivering novel chemotherapies such as oligonucleotides. For example, AS1411 aptamers that target nucleolin can be conjugated to gold nanoparticles [153]. These conjugated gold nanoparticles can breach the BBB [153]. Another study embedded paclitaxel into nanoparticles prepared with emulsifying wax (cetyl alcohol/polysorbate 60 in a 4:1 *w*/*w* ratio) used as the oil phase, water, and Brij 78 as the surfactant [154]. Wax nanoparticles led to enhanced brain uptake of paclitaxel in a rat model [154]. Biodegradable nanomaterials could also aid in delivering therapeutics to resection sites in glioblastoma with sustained drug release [155]. Investigating the ability of diverse nanomaterial-delivery systems to cross the BBB to treat glioblastoma and for sustained drug release offers hope for the development of efficacious therapeutics.

## 18. Conclusions

**Figure 1:**
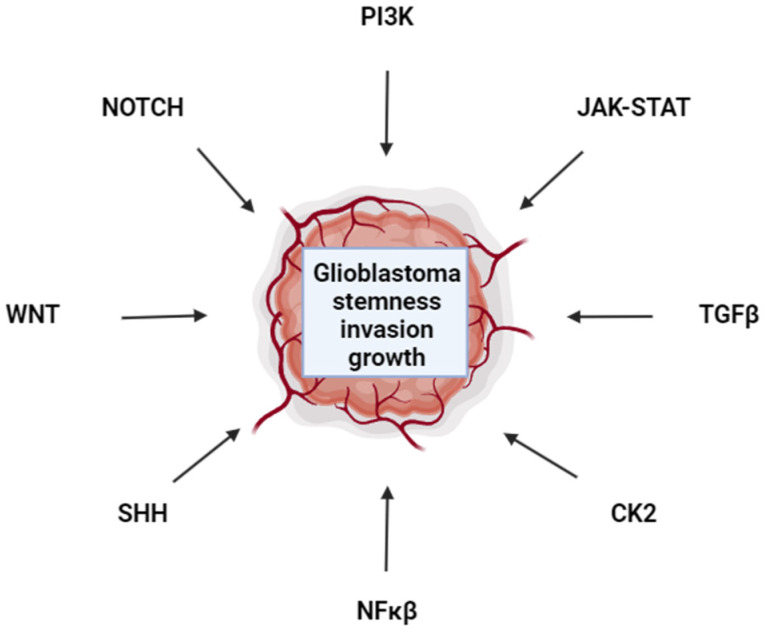
Glioblastoma Signal Transduction Pathways. Pathways that contribute to glioblastoma are indicated. The interconnectedness, and redundancy of these pathways in promoting glioblastoma remain to be fully elucidated and are active areas of research.

Despite scores of clinical trials to identify efficacious interventions for glioblastoma, the prognosis remains poor for this aggressive cancer [78]. Barriers at the forefront of this field include overcoming the BBB, TTFields, oncolytic viruses, and novel targeted therapeutic strategies such as FOXO1 and LIF inhibition [36,75,112,145]. Feedback mechanisms that render targeted therapies ineffective drive the need for the development of combination therapies that target oncogenes and functionally redundant mechanisms that drive glioblastoma [72]. It is striking that diverse developmental pathways all converge to promote glioblastoma stemness, invasion, and proliferation (Figure 1). How these diverse pathways interact to promote glioblastoma phenotypes needs to be more fully delineated. Understanding glioblastoma plasticity to target numerous cell types found in tumors will be vital to treating this cancer [156]. If the stem niche is inhibited, perhaps cells adopt a more differentiated state that is fluid and can de-differentiate back to a stem phenotype once inhibitors are no longer present. Therefore, targeting the spectrum of cell fate types within a tumor may be necessary. The more that is understood about cell fate specification in glioblastoma, the better targeted therapy combinations can be rationally designed. Furthermore, as precision medicine comes online, patients will more quickly receive therapies designed to match their specific tumor landscape to improve treatment efficacy.

Treating glioblastoma is especially difficult given the BBB. Nanoparticle delivery systems and development of other novel delivery systems such as drug-embedded wafers inserted at the surgical site and intra-tumor injections hold promise for progress in treating glioblastoma [155]. Even if the drug delivery barrier for glioblastoma is solved, finding therapeutics that induce apoptosis in these cells is challenging. As our understanding of glioblastoma signal transduction pathways becomes more sophisticated, refinements in combination therapies may foster innovations to improve prognosis, providing needed advances to treat these devastating cancers.

## Figures and Tables

**Table 1:** EGFR inhibitor clinical trials for glioblastoma.

Drug Name	Context	Impact
Gefitinib	Human glioblastoma clinical trial	Minimal to no activity [56]
Erlotinib	Human glioblastoma clinical trial	Minimal to no activity [58]
Afatinib	Human glioblastoma clinical trial	Minimal to no activity [55]
Lapatinib	Human glioblastoma clinical trial	Minimal to no activity [57]
Osimertinib	Human glioblastoma clinical trial	Minimal to no activity [53]
Cetuximab	Human glioblastoma clinical trial	Minimal to no activity [59]
Rindopepimut	Human glioblastoma clinical trial	Minimal to no activity [54]
Depatuxizumab mafodotin + TMZ	Human glioblastoma clinical trial	Minimal to no activity [52]
